# Accelerated wound healing phenotype in Interleukin 12/23 deficient mice

**DOI:** 10.1186/1476-9255-8-39

**Published:** 2011-12-20

**Authors:** Marie AT Matias, Jodi M Saunus, Saso Ivanovski, Laurence J Walsh, Camile S Farah

**Affiliations:** 1 University of Queensland, School of Dentistry, Brisbane, Australia; 2 University of Queensland, University of Queensland Centre for Clinical Research (UQCCR), Brisbane, Australia; 3 Griffith University, School of Dentistry and Oral Health, Gold Coast, Australia

**Keywords:** Wound healing, Interleukin-12, Interleukin-23, p40, Inflammation, Angiogenesis

## Abstract

**Background:**

The concept that a strong inflammatory response involving the full complement of cytokines and other mediators is critical for unimpaired healing has been challenged by wound healing studies using transgenic and knockout (KO) mice. The present study explored the effect of abrogation of the p40 subunit, which is shared by the pro-inflammatory cytokines interleukin (IL)-12 and IL-23, on wound closure of excisional oral mucosal wounds.

**Methods:**

Double IL-12 and IL-23 KO mice and C57BL ⁄ 6J wildtype mice were wounded on the dorsal surface of the tongue using a 2 mm biopsy punch. The degree of epithelialization was examined histologically. At specific timepoints wounds were examined for cellular and molecular markers for inflammation and angiogenesis using 1) immunohistochemistry; 2) analysis of RNA expression; and 3) flow cytometric analysis.

**Results:**

Compared to wild type controls, KO mice displayed enhanced healing, which was driven by a greater influx of neutrophils and macrophages during the early stages of wound healing, and increased induction of messenger RNA (mRNA) for endothelial derived neutrophil attractant (ENA78) chemokine and macrophage inflammatory protein-2 alpha (MIP-2α). Increased mRNA for monocyte-attracting chemokines including monocyte chemoattractant protein (MCP)-1 and MCP-3 was seen from day 1, together with higher levels of IL-1β and IL-6 within 24 hours after wounding. In addition, mRNA for vascular endothelial growth factor (VEGF)-A was upregulated in KO mice within 2 hours after injury, and higher expression of this mediator was confirmed by immunohistochemistry.

**Conclusion:**

Overall, the accelerated oral mucosal wound healing seen in IL-12/IL-23p40 KO compared to wildtype mice was associated with the early establishment of an inflammatory response and vascularization.

## Background

A wound undergoes three distinct stages which overlap in time as it heals: inflammation, proliferation and remodeling/tissue maturation. The characteristics of the inflammatory response define the progress of a healing wound. For example, diabetic ulcers and chronic pressure ulcers are associated with persistent inflammation [1], while keloids or scar formation is rarely seen in fetal wounds which show a diminished inflammatory response [2]. Studies using transgenic and knockout (KO) mice shed significant light on the cellular and molecular mechanisms in wound healing. For example, PU.1-knockout mice which are deficient in neutrophils and macrophages show slightly enhanced rates of re-epithelialization, enhanced angiogenesis, and an absence of fibrosis [3], with phagocytosis being undertaken by fibroblasts. A cluster of genes expressed after wounding has been linked with tissue repair genes, and another with inflammation and its consequences. The former provides the basic repertoire to allow normal healing to occur, even in the absence of professional phagocytes [4]. This gene cluster concept cast doubts on the dogma that inflammation is mandatory for repair after injury.

Wound healing studies in cytokine KO mice have shown that both pro- and anti- inflammatory cytokines influence the healing process. While IL-6 KO mice [5] and IL-1 receptor antagonist (IL-1ra) KO mice [6] show slower healing, mice which are deficient in TNF receptor p55 [7] or IFN-γ show accelerated healing, most likely by augmenting TGF-β1 mediated signalling pathways [8]. A recent study of wound healing in IL-10 KO mice also showed accelerated wound healing [9]. IL-10 down-regulates several pro-inflammatory cytokines including IL-1, IL-6, IL-12, IFN-γ and TNF-α. *IL-10^-/- ^*mice show accelerated re-epithelialization as well as greater macrophage infiltration and enhanced wound contraction compared to wild-type controls [9].

Dissecting the process of wound healing using other well established cytokine KO mice such as IL12/IL-23p40 is of interest because it is shared by two inflammatory cytokines, interleukin-12 (IL-12) and interleukin-23 (IL-23). IL-12/IL-23p40 is produced primarily by activated inflammatory cells such as macrophages, neutrophils and dendritic cells as well as by keratinocytes and respiratory epithelial cells [10-12]. The effects of IL-12 and IL-23 are related but distinct. IL-12 promotes differentiation of CD4+ naïve T cells to TH_1 _effector cells which stimulate natural killer (NK) cells and CD8+ T cells to produce IFN-γ [11]. In contrast, IL-23 stimulation of naïve CD4+ T cells in conjunction with IL-1β gives rise to TH_17 _cells, which secrete multiple cytokines including IL-17A, IL-17F, IL-22, IL-26, IFN-γ, IL-6 and TNF-α. There is also evidence that the IL-23-17A axis is important in early mucosal immune responses [13].

A potential role for IL-12 and IL-23 in wound healing is suggested by IL-12 having anti- angiogenic activity which is mediated through its effects on promoting secretion of IFN-γ [14], which in turn increases production of IFN-γ-inducible protein 10 (IP-10) a potent inhibitor of angiogenesis which prevents formation of new blood vessels [15-17]. The IL-12/IL-23p40 molecule itself appears to be a natural antagonist to IL-12, competitively binding to the IL-12β1 receptor [18,19]. The molecule also serves as a chemoattractant for macrophages [20] and it promotes migration of activated dendritic cells [21]. Blockade of IL-12/IL-23p40 through its effects on IL-23 may also influence wound healing by up-regulation of MMP-9 which has downstream effects on angiogenesis [22]. Despite the above work, the basic question of whether inflammation is beneficial or a hindrance in the wound repair process remains, and use of p40 KO mice may provide further insight into this. The present study uses an oral mucosal wound healing model based on excisional tongue biopsies which follows wound healing events as in the skin.

This study is the first to report that abrogation of the IL-12/IL-23p40 molecule results in accelerated re-epithelialization of wounds, and reveals that IL-12 and IL-23 influence wound healing by modulating early inflammatory responses and subsequent angiogenesis.

## Methods

### Animals

Specific pathogen-free female *IL-12/IL-23p40^-/- ^*mice and their respective C57BL/6J controls 6-8 weeks of age were used in these experiments. *IL-12/IL-23p40^-/- ^*mice raised on C57BL/6J background [23] were obtained from the Monash Institute of Reproduction and Development, Monash University Melbourne Australia. Mice were bred at Herston Medical Research Centre, Brisbane, Australia, with regular polymerase chain reaction (PCR) based genotyping. Mice were housed in filter-top cages in a PC2 facility, and provided food and water *ad libitum*. Animal experiments were approved by the Animal Experimentation Ethics Committee of the University of Queensland, and carried out in accordance with the National Health and Medical Research Council's Australian Code of Practice for the Care and Use of Animals for Scientific Purposes, 1997.

### Wound healing model

Under light general anesthesia, a wound was created using a standard 2 mm diameter punch biopsy positioned along the dorsal mid-line of the tongue, 3 mm from its anterior tip. Animals were sacrificed at specific timepoints for: 1) immunohistochemistry; 2) analysis of RNA expression; and 3) flow cytometric analysis. In all cases, a 4 mm punch biopsy was used to harvest the wound site so that the wound was removed *in toto*. For analysis of RNA expression, harvested wounds were stored immediately in RNA*later*^® ^*(Ambion, Applied Biosystems, Austin, TX, USA) *and kept at -80°C. Samples harvested for histology were fixed in 4% paraformaldehyde in phosphate-buffered saline (PBS) for a minimum of 2 days, then bisected and embedded in paraffin. Serial sections from the central portion of the wounds were used for histological analysis. For flow cytometric analysis, tissues were placed into RPMI 1640 medium *(Invitrogen; Mulgrave, VIC*, *Australia) *containing antibiotics/antimycotics *(Ab/Am; Gibco^®^-Invitrogen, Mulgrave, VIC, Australia) *and processed immediately.

### Histology

For each strain of mice and for each time point, 10 mice were wounded and wounds were harvested on day 0 (unwounded) and at 1, 4, 7 and 10 days after wounding. Serial sections from the central portion of the wound were stained with haematoxylin and eosin, and the extent of wound closure determined. A wound was defined as completely healed/closed when all central serial sections demonstrated an intact superficial epithelial layer over the wound area.

### Histochemistry

Harvested wounds (n = 3) from *IL-12/IL-23p40^-/- ^*mice and C57BL/6J controls were collected at day zero and at 1, 4 and 7 days after wounding. Serial sections from the central wound area were used for analysis. Antigen retrieval from the fixed tissues was undertaken using microwave treatment in 0.01 M sodium citrate buffer. Prior to the first layer reagent, sections were treated with Peroxidazed 1 *(Biocare Medical, LLC; Concord, CA) *for 10 minutes to block endogenous peroxidise activity, and then incubated with normal donkey serum (5%) for 20 minutes to stop non-specific binding. The first layer anti-mouse antibodies were applied at pre-determined optimal dilutions overnight. Markers for neutrophils (*AbD, Serotec, Oxford, UK*), macrophages *(M3/84) (BD Pharmingen, Franklin Lakes, NJ, USA)*, CD31 *(Santa Cruz Biotechnology, Santa Cruz, CA, USA) *and Factor VIII related antigen *(Zymed Laboratories, San Francisco, CA) *were used. The secondary antibody (*SuperPicture Broad Spectrum, Invitrogen, Mulgrave, VIC, Australia*) was then applied and incubated for 1 hour. For visualization, the Vector NovaRed Substrate Kit (*Vector Laboratories, Burlingame, CA*) was used. Sections were counterstained with haematoxylin prior to mounting. For Factor VIII staining, trypsin digestion *(Zymed Digest-All, Zymed Laboratories Inc., San Francisco, US) *was performed for 1 hour at room temperature before applying the primary antibody. The slides were then scanned using Aperio Scanner (Aperio, California, USA) and the digital slides stored for analysis. For each specimen, equivalent fields were examined on serial sections, thus enabling comparisons to be made between markers. In all cases, localization was assessed both qualitatively and quantitatively. The number of neutrophils and macrophages was calculated as number of positive cells counted in 10 high-power fields *(× 400 magnification) *divided by 10 [24,42]. Anti-CD31 antibody was used to localize areas of angiogenesis. This antibody stains cell surfaces of monocytes, neutrophils, platelets and endothelial cells, and has been used frequently as a marker of angiogenesis in a number of wound healing studies[7,9,25,26]. Using Aperio ImageScope v10 software area for CD31 and Factor VIII staining was measured. As previously described, CD31 positive areas within the wound bed were measured and the percent vascularization was calculated as: % CD31+ve area = (CD31+ve area/Total wound bed area) × 100% [25]. Anti-Factor VIII, a marker which is specific for endothelial cells, megakaryocytes and platelets was used to confirm areas of angiogenesis. The area of Factor VIII staining was calculated as % Factor VIII (Angiogenesis) = (Factor VIII+ve area/Total wound bed area) × 100%.

### Analysis of RNA expression

RNA was isolated from 2 mice per strain (*IL-12/IL-23p40^-/- ^*mice and C57BL/6J) at four timepoints: day 0 (unwounded), 2 hours, 1 day and 4 days after wounding, from the 4mm biopsies using TRIZOL (*Invitrogen, Mulgrave, VIC, Australia*) according to the manufacturer's instructions. To prevent amplification from genomic DNA templates, total RNA was DNAse- treated using DNA-free™*(Ambion, Applied Biosystems, Austin, TX, USA) *according to the manufacturer's instructions. RNA concentration was determined by spectrophotometry using the NanoDrop^® ^ND-1000 *(Thermo Scientific, Washington, USA)*, and the ratio of absorbance at 260 nm and 280 nm was used to assess the purity of the sample. To check RNA integrity, total RNA was electrophoresed in 1.2% agarose gel. 28S and 18S rRNA served as markers for RNA integrity.

cDNA was prepared using the "Superscript III" kit (*Invitrogen, Mulgrave, VIC, Australia*) using random hexamers *(Promega, Alexandria, NSW, Australia) *from 3 μg of total RNA according to the manufacturer's instructions. mRNA for 84 genes involved in inflammation and immunity including IL-1B, IL-6, MCP-1, MCP-2, Cxcl2 (MIP-2) and Cxcl5 (Ena-78) was quantified in triplicate using the SuperArray RT^2^Profiler™ PCR Array (PAMM-073E) *(SABioscience, Frederick, MD, USA)*. Fold changes in expression of the genes of interest (GOI) between samples were calculated using the software provided by the array manufacturer. The average of housekeeping genes (HKG) including glucoronidase β-1 (Gusb), hypoxanthine guanine phosporibosyl tranferase 1 (Hprt1), heat shock protein 90 kDa α class B member 1 (Hprt1), glyceraldehyde-3-phosphate dehydrogenase (Gapdh) and Actin-β 1 (Actb) was used. The change in C_t _value was calculated as: $\Delta {\text{C}}_{\text{t}}={\text{C}}_{\text{t}}^{\text{GOI}}-{\text{C}}_{\text{t}}^{\text{AVG HKG}}$. The change for each gene across two groups was calculated as: ΔΔC_t _= C_t_(group 1) - C_t_(group2) where group 1 was the control and group 2 the experimental group.

Following this, the fold-change for each gene from group 1 to group 2 was calculated as 2(-ΔΔC_t_) with *p-value *of 0.05.

VEGF-α mRNA and the loading control 18S ribosomal RNA (rRNA) were quantified on a 7900 Sequence Detection System in triplicate using SDS 2.2.3 software *(Applied Biosystems, Mulgrave, VIC, Australia)*. Reactions contained SYBR green PCR master mix *(Applied Biosystems, Mulgrave, VIC, Australia)*, VEGF-α primer (F-cacgacagaaggagagcaga, R-aagatgtccaccagggtctc, 100 nM) and 18S (F-catttggagggcaagtctgg, R-tcccaagatccaactacgagc, 100 nM) and the template diluted appropriately in distilled water. Cycling conditions were 10 min @ 95°C, followed by 45 cycles of 15 sec @ 95°C and 1 min @ 59°C. Complementary DNA synthesis reactions conducted with no reverse transcriptase were included to control for genomic DNA contamination. Post-PCR melt analysis was conducted to ensure that the quantified PCR products were pure and free of non-specific amplicons. The comparative ΔΔC_t _method *(Applied Biosystems, Mulgrave, VIC, Australia) *was used to determine cytokine mRNA expression relative to 18S rRNA, relative to unwounded wildtype. Fold changes were calculated as mentioned above.

### Flow cytometry

Six tissue samples were harvested and pooled for each of *IL-12/IL-23p40^-/- ^*and C57BL/6J mice for the following timepoints: day 0 (unwounded), 2 hours, 1 day, 4 days and 7 days after wounding. Tissues were homogenized and a single cell suspension prepared for flow cytometry analysis as described previously [13]. Briefly, tissues were placed in digestion medium [RPMI-1640 (*Invitrogen, Mulgrave, VIC, Australia*), 5% fetal calf serum (FCS) *(Gibco^®^-Invitrogen, Invitrogen, Mulgrave, VIC, Australia*), Ab/Am *(Gibco^®^-Invitrogen, Mulgrave, VIC, Australia)*, monensin *(eBioscience; San Diego, CA)*, collagenase 1A (2 mg/mL; *Sigma-Aldrich; Castle Hill, NSW, Australia*) and hyaluronidase (100U mL^-1^; *Sigma; Castle Hill, NSW, Australia*)] after being minced finely using a scalpel blade. The samples were incubated in digestion medium for 1.5 hours at 37°C in a shaking water bath. Homogenates were then filtered through a metal filter (250 μm) and the residue resuspended in Cell-free Dissociation buffer *(Life Technologies, Invitrogen, Mulgrave, VIC, Australia*). The collected single cell suspension was then diluted in RPMI-1640 containing 5% FCS, and cells then washed by centrifugation at 400 *g *for 3 min at 4°C. Cells were then resuspended in staining buffer [Flow Cytometry Staining Buffer *(eBioscience; San Diego, CA)*, containing 2% FCS *(Gibco^®^-Invitrogen, Mulgrave, VIC, Australia*)] to a final concentration of 4 × 10^7 ^cells/mL.

For each immunofluorescence reaction, 2 × 10^6 ^cells were incubated in 50 μl blocking buffer [1 μg anti-CD16/32 in 50 μl uL of staining buffer] for 10 minutes on ice prior to incubation with monoclonal antibodies (mAb) at pre-optimized dilutions as follows: fluorescein isothiocyanate-conjugated (FITC) anti-F4/80 mAb (macrophages) (1 μg per 2 × 10^6 ^cells, *(eBioscience; San Diego, CA)*, phycoerythrin (PE) conjugated anti-neutrophil mAb (20 μl per 2 ×10^6 ^cells, MCA771PE; *Abd Serotec, Raleigh, NC*) and allophycocyanin (APC) conjugated anti-CD31 mAb (1 μg per 2 × 10^6 ^cells, *(eBioscience; San Diego, CA)*, for 30 minutes in the dark at 4°C. Samples were washed three times by centrifugation in PBS at 400 *g *for 5 minutes at 4°C, then the cells resuspended in Cell Dissociation Buffer *(Life Technologies, Invitrogen, Invitrogen, Mulgrave, VIC, Australia*) for flow cytometric analysis within 2 hours using an LSRII flow cytometer *(Becton Dickinson, North Ryde, Australia)*. Data collection was based on at least 10,000 events. Data were analyzed using FACSDiva software *(V6.0; Becton Dickinson, North Ryde, Australia)*. Gates were established using unstained control samples including a 1:1 mixture of cells from C57BL/6J mice and *IL-12/IL-23p40^-/- ^*mice.

### Statistical Analysis

To test the null hypothesis that there was no difference between healing in test and control mice, the log-rank (Mantel-Cox) test was used. Differences in gene expression and in histological markers (inflammatory infiltrate, vascularity and granulation tissue) were assessed using the *t- test*.

## Results

### Accelerated rate of oral mucosal wound healing in IL-12 and IL-23 deficient mice

**Figure 1:**
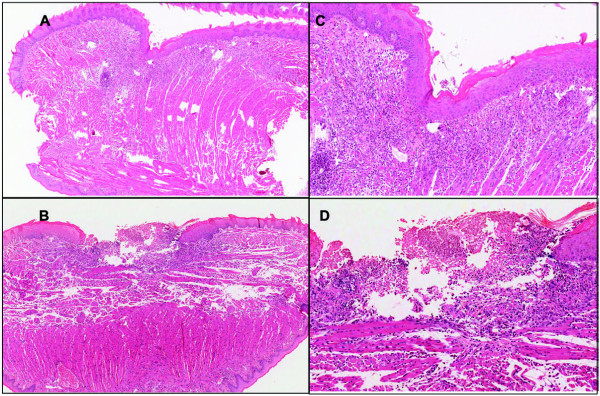
**Day 4 after wounding, complete wound closure was observed in the IL-12/23p40^-/- ^mice (Figure 1A and 1B) compared with open wounds (incomplete epithelialization) in the control group**. Original magnification ×4 (Figures 1A and 1C) and ×10 (Figures 1B and 1D).

Histological analysis of healing wounds in *IL-12/IL-23p40^-/- ^*and C57BL/6J showed an accelerated healing pattern in the KO mice compared to WT mice (Log-rank test, *p = 0.002*; Table 1), based on 10 mice (1 wound per mice) from each group examined at 4 and 7 days after injury. Wound closure was defined as complete re-epithelialization from all central sections of a wound. All wounds from both strains of mice showed partial epithelial coverage at 1 day after wounding. At 4 days (Figure 1), 40% of wounds showed complete wound closure in the *IL-12/IL-23p40^-/- ^*mice, compared with only 10% in the WT counterparts. By 7 days after wounding, some 90% of wounds in the *IL-12/IL-23p40^-/- ^*mice had healed, compared with only 40% in the WT mice. By 10 days, all wounds in the KO mice showed an intact epithelial layer over the wound bed, while in the wildtype mice 1 of the 10 mice still had yet to establish complete epithelial cover at the same time point.

**Table 1:** Percentage of complete wound closure in IL-12/23p40^-/- ^and C57BL/6J mice.

Time after wounding	Day 1	Day 4	Day 7	Day 10
IL-12/23p40^-/-^	0%	40%**	90%**	100%

C57BL/6J	0%	10%	40%	80%

Wounds from the IL-12/23p40^-/- ^mice healed significantly faster at days 4 and 7 compared to C57BL/6J control mice. Log-rank (Mantel-Cox) Test, p = 0.002**; n = 10 per strain, per time point.

### Early inflammatory response cell infiltrate in knock-out mice

**Figure 2:**
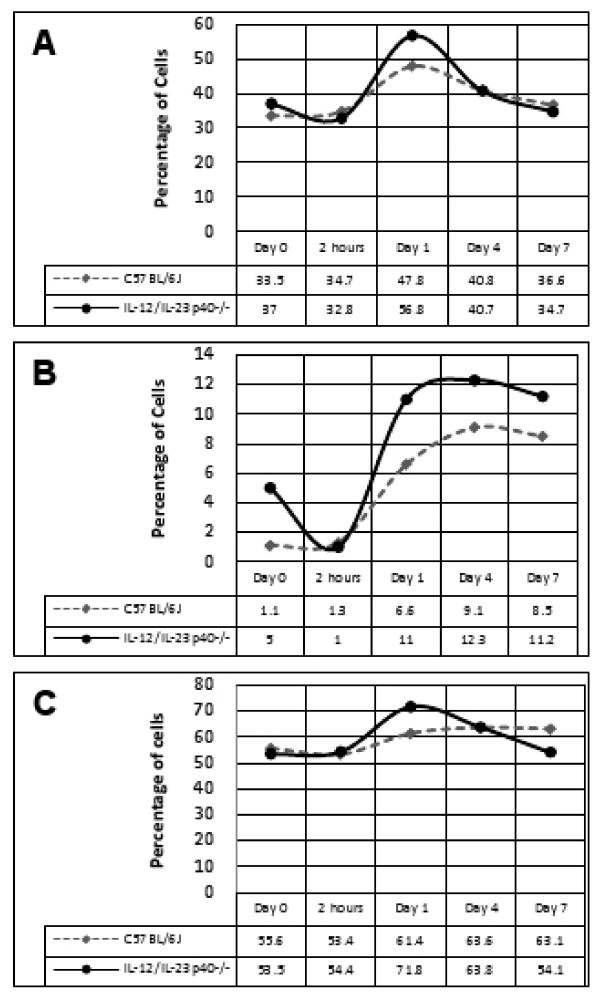
**Percentage of neutrophils (A), macrophages (B) and CD31 positive cells (C) detected in healing wounds of IL-12/IL-23p40^-/- ^mice compared with C57BL/6J per 10,000 events**.

The nature of the cellular infiltrate in the healing wounds was assessed using flow cytometry using markers for macrophages, neutrophils and CD31 positive cells (Figure 2). More infiltrating neutrophils were detected 1 day after wounding in the KO mice, however the increase did not reach statistical significance. KO mice displayed an increase in macrophage numbers from 0 to 7 days after injury, with almost double at 1 day after wounding compared to WT mice (11% vs. 6.6%, respectively). There was a small increase in CD31 positive cells at day 1 in the KO mice, but this decreased to baseline levels by day 7. An opposite trend was seen in the WT counterparts, where the number of CD31 positive cells increased from day 1 to day 7 after wounding. Overall there was a consistent trend for higher numbers of neutrophils, macrophages and CD31 positive cells in KO wounds.

Analysis of the neutrophil and macrophage infiltrate in the wounds using immunohistochemistry confirmed the trend of a greater inflammatory infiltrate in the KO mice There was a trend for more infiltrating neutrophils in KO mice 1 day after wounding compared to wildtype mice with the reverse trend at 4 days after wounding. Likewise, there was a trend for more infiltrating macrophages at days 0, 1 and 4 in the KO mice compared with controls, which was in keeping with the flow cytometry data.

### Early upregulation mRNA of inflammatory and immune markers

The summary of fold changes of all genes studied is provided in additional file 1, table S1. Genes of interest are shown in Table 2. There were significant increased levels (more than 2 fold increase, *p < 0.05*) within 24 hours after wounding in *IL-12/IL-23p40^-/- ^*mice for the chemokines; endothelial derived neutrophil attractant (ENA78), macrophage inflammatory protein-2 alpha (MIP-2α), monocyte chemoattractant protein (MCP)-1 and MCP-3. MCP-1 and MCP-3 mRNA induction occurred from day 1 after wounding and peaked at day 4. The early induction of these chemokines was followed by reduction to baseline levels by day 7. In addition, the pro-inflammatory cytokines IL-1β and IL-6 showed a marked increase 1 day after wounding in KO mice, with fold increases of 953× and 247× respectively, compared to 165× and 20× in the WT counterparts (Table 2). This rapid increase was followed by an equally rapid return to baseline levels by day 4. Overall, the data support an early inflammatory response to wounding in the KO mice compared to WT mice.

**Table 2:** Fold changes of genes of interest during the time-course of a healing wound in IL-12/23p40^-/- ^and C57BL/6J mice.

Gene of Interest	Strain of Mouse	Fold-Change		
		
		Day 1	Day 4	Day 7
	IL-12/IL-23p40	71.59	9.62	7.92
	
**MCP-1**	C57BL/6J	27.21	6.02	5.16

	IL-12/IL-23p40	128.6	17.63	11.77
	
**MCP-3**	C57BL/6J	58.07	8.76	8.55

	IL-12/IL-23p40	11235	788.3	1104
	
**MIP-2**	C57BL/6J	2073	763.9	274.5

	IL-12/IL-23p40	3028	1296	1523
	
**ENA-78**	C57BL/6J	974.4	2037	821.8

	IL-12/IL-23p40	953.9	86.42	215.6
	
**IL-1β**	C57BL/6J	165.0	115.7	59.11

	IL-12/IL-23p40	247.1	24.59	14.54
	
**IL-6**	C57BL/6J	20.84	35.77	13.76

Expression profile of chemokines (MCP-1, MCP-3, MIP-2 and ENA-78) and cytokines (IL-1β and IL-6) seen in the IL-12/IL-23p40^-/- ^mice parallel the early recruitment of inflammatory cells and early establishment of an inflammatory response. Fold-changes were calculated using C57BL/6J unwounded samples as control group. Student's t-test, *p value *<*0.05*; n = 2 per strain, per time point.

### Early establishment of angiogenesis in IL-12/IL-23p40^-/- ^mice

**Figure 3:**
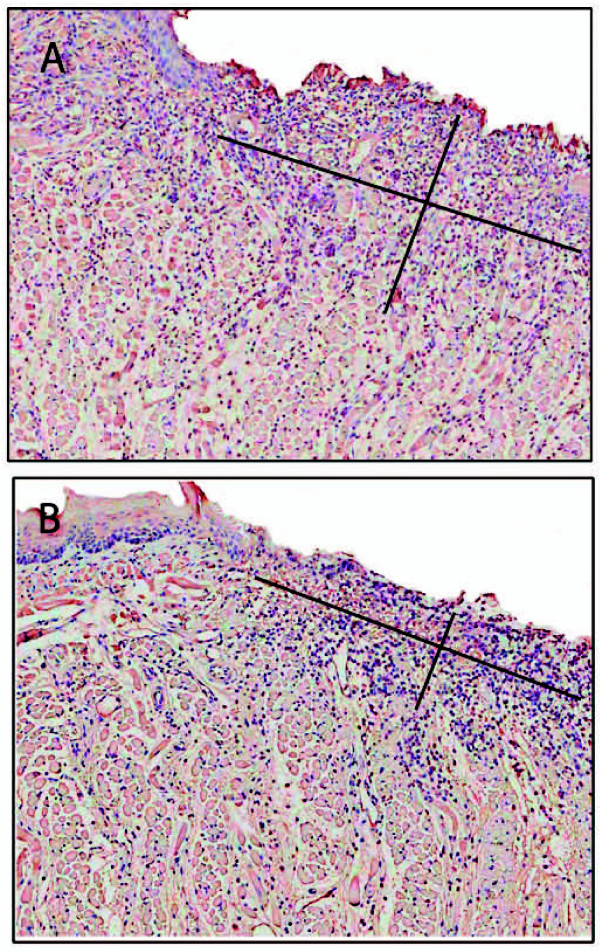
**Analysis of angiogenesis at wound sites**. Sections were stained with anti-CD31 antibody. Immunohistochemical sections 1 day after wounding in IL-12/IL-23p40^-/- ^mice (A) and C57BL/6J (B). The area of CD31^+ ^site was significantly larger in the KO group compared to control group 1 day after wounding (55.3 ± 6.7 vs. 27.3 ± 4.6; *p = 0.026*). The diagonal cross overlaid on the slide image shows approximately areas of CD31^+ ^sites.

**Figure 4:**
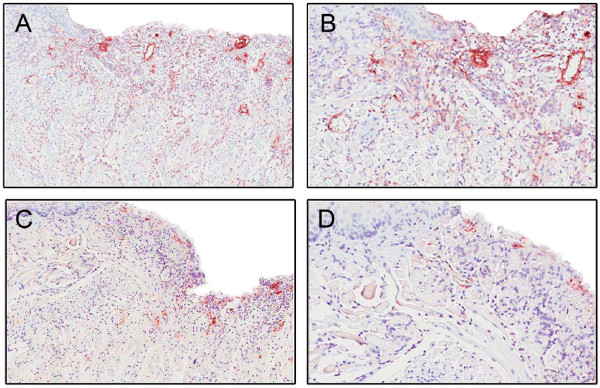
**Analysis of angiogenesis at wound sites**. Figures 4A and 4B show immunohistochemical sections 1 day after wounding in IL-12/IL-23p40^-/- ^mice. Sections were stained with anti-Factor VIII. Original magnification ×4 and ×20, respectively. Figures 4C and 4D show sections of C57BL/6J wounds 1 day after wounding (stained with anti-Factor VIII). Original magnification ×4 and ×20, respectively. There was a significantly larger Factor VIII positive area in the knockout mice compared to wild-type controls (*t-test; p value 0.008)*.

**Figure 5:**
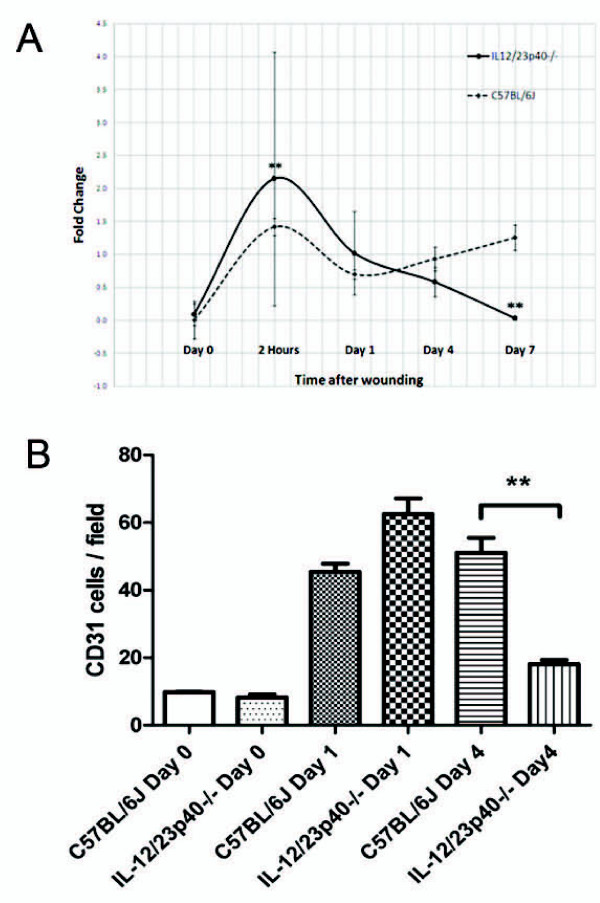
**Establishment of angiogenesis: Figure 5A demonstrates VEGF-A was significantly up- regulated at 2 hours after wounding in the knockout group compared to the control group.** At day 7 after wounding, VEGF-A expression between test and control groups was significantly different, with VEGF-A expression in the knockout group returning to baseline levels, and in the control group appearing to have increased. Figure 5B demonstrates CD31^+ ^cell recruitment into wound sites. The number of CD31^+ ^cells recruited per high-power microscopic fields counted. Data is expressed as the mean ± SEM (n = 3). There was a higher number of CD31^+ ^cells observed 1 day after wounding in the knockout mice compared to control group. This was not statistically significant. There was a statistically significant difference in the number of CD31^+ ^cells at day 4 after wounding between test and control group (*t-test; p value 0.002)*.

Anti-CD31 antibody was used to estimate the extent of angiogenesis. A significantly larger area of vascularization over the wound bed was demonstrated in KO mice at day 1 compared to WT mice (55.3 ± 6.7 vs. 27.3 ± 4.6; *p = 0.026*) (Figure 3). Anti-Factor VIII was used to confirm areas of angiogenesis. The results from Factor VIII immunostaining were consistent with those for CD31,

with a greater area of staining at day 1 in the KO mice than in the WT mice (25.3 ± 3.0 vs. 9.7 ± 1.2*; p = 0.008*) (Figure 4). These two observations complemented the up-regulation of mRNA levels for VEGF-A in the KO group which was seen 2 hours after wounding compared to the WT mice. By 7 days after wounding, VEGF-A levels in the KO mice had returned to baseline, but they remained elevated in the WT mice *(p < 0.05*) (Figure 5A).

Cell counts for CD31 showed a larger infiltrate at day 1 in the KO mice compared to the WT mice (Figure 5B). There was a statistically significant difference in CD31 positive stained cells 4 days after wounding. The control group had an increasingly larger infiltrate, while there was a decreased number of CD31^+ ^cell infiltrate in the KO group (*p = 0.002)*. This observation between the two strains of mice at the same time point was clearly visible at the base of the wounds, adjacent to the wound edges. In the KO mice, the base of the wound displayed a more organized granulation tissue compartment compared to the WT mice where numerous blood vessels are scattered around a largely unorganized matrix compartment.

## Discussion

Research into wound healing can help decipher the intricate molecular and cellular mechanisms involved in repair, and can also provide clues to novel therapeutic targets for promoting regenerative healing. This preliminary study investigated the effect of deficiency of IL-12 and IL-23 in wound healing using cytokine KO mice. This study is the first to demonstrate that *IL-12/IL-23p40^-/- ^*mice display significant acceleration of healing of oral mucosal wounds compared to controls. The accelerated wound closure likely occurs through multiple mechanisms. Firstly, there is a small influence on neutrophil infiltration at day 1 after wounding in the KO mice but is likely to be of lesser importance than the enhanced influx of macrophages which follows it. IL- 12/IL-23p40 is produced by both neutrophils and macrophages as well as by other inflammatory cells, and is a known chemoattractant for macrophages, however its effects are less potent than other macrophage chemoattractants such as MIP-1, MCP-1 and RANTES [27-29]. Because of redundancy, the absence of the IL-12/IL-23p40 molecule in KO mice may elicit a greater compensatory response from other pathways which enhance the recruitment of macrophages. Together, the increased infiltration of neutrophils and macrophages appears to have a favorable effect on the progression of the healing process.

Preliminary analysis of mRNA levels of chemokines which are chemoattractants for neutrophils and macrophages partially explains the trend for a larger inflammatory infiltrate in the KO group. Both GRO-1 and ENA-78 are released by degranulating platelets and initiate neutrophil recruitment into the wound bed [30-32]. The early induction of these chemokines after wounding in IL-12/IL-23p40 KO mice would enhance neutrophil influx. Likewise, enhanced expression of MIP-3a and MDC may also contribute to earlier recruitment of macrophages in the KO mice. MIP-3a and MDC mediated recruitment of macrophages augments the inflammatory response and promotes debridement during wound healing [27,33]. In addition, the chemokines GRO-1, MIP-2 and ENA-78 mediate angiogenesis and epithelialization [33,34]. Early induction of such chemokines would create an environment conducive to accelerated epithelialization and formation of capillaries in the healing wound.

Polymorphonuclear leukocytes and macrophages as well as some resident fibroblasts and keratinocytes secrete IL-1β and IL-6 in a healing wound, and mRNA expression of these cytokines is up-regulated during the inflammatory phase of healing [35-37]. Both IL-1β and IL-6 showed rapid and marked increase within 1 day of wounding in KO mice. IL-1 stimulates keratinocyte migration and proliferation, and enhances fibroblast secretion of fibroblast growth factor (FGF)-7 [32]. IL-6 is chemoattractive to neutrophils, and a potent mitogen for keratinocytes. The marked upregulation of IL-1β and IL-6 provides evidence of an early inflammatory response, but most importantly this was followed by rapid return to baseline levels which demonstrate a robust but controlled inflammatory response. The simultaneous presence of IL-6 and IL-1 within 24 hours after wounding would increase neutrophil infiltration into the wound site and promote epithelial closure, and thereby contribute to accelerated healing [32].

Early induction of VEGF-A is a further mechanism which would be operating in KO mice. This growth factor promotes the early events in angiogenesis, particularly endothelial cell migration and proliferation [38-44], which in turn leads to the formation of granulation tissue. Angiogenesis is essential for effective healing, and inhibition of angiogenesis delays or impairs wound healing [45,46]. In the *IL-12/IL-23p40^-/- ^*mice, angiogenesis was well established by the first day after wounding, as demonstrated by staining for CD31 and Factor VIII in tissue sections, and by analysis of cell homogenates for CD31 positive cells. The earlier establishment of an angiogenic response in the KO mice is implicated as a major mechanism which underpins accelerated wound healing.

As mentioned previously, the influence of IL-12 on angiogenesis is mediated through IFN-γ [14], however recent findings support an alternative pathway. The presence of IL-12 has been associated with down-regulation of MMP-9 [15,47-49] which directly affects endothelial cell function and inhibits angiogenesis [49]. MMP-9 facilitates angiogenesis by breaking down the ECM, allowing endothelial cells to migrate, proliferate and differentiate into new capillaries [50]. Additionally, MMP-9 may further support this process by releasing matrix-sequestrated angiogenic growth factors, such as PDGF [51]. Recently, evidence of a direct link between MMP-9 and VEGF-induced angiogenesis has also been demonstrated [52]. The findings from this study infer that deficiency of IL-12 appears to be influential in facilitating the establishment of an early angiogenic response to wounding. Whether this effect is mediated through IFN-γ or through the downstream effect of MMP-9 on angiogenesis is yet to be determined. Nevertheless, the reciprocal relationship between IL-12, MMP-9 and VEGF warrants further investigation in light of new findings in this study.

Taken together, the expression profiles for chemokines and cytokines in this preliminary analysis in IL-12/IL-23p40^-/- ^mice parallel the key events of early recruitment of phagocytes, enhanced epithelialization, and early establishment of angiogenesis which together contribute to the favorable healing phenotype observed. The next aspect of this study would involve further investigation and confirmation of causal roles of mediators identified including specific chemokines, as well as facilitators of angiogenesis. In addition, experiments including single KO mice lacking only IL-12 or IL-23 would be used to distinguish the contribution of this distinct cytokines to the accelerated healing phenotype observed in this study. Apart from using these mediators as potential novel therapeutic agents of repair, this study also provides a platform to investigate related chronic conditions such as impaired healing in diabetics, as well as cutaneous conditions such as psoriasis. The relationship of IL-12 and IL-23 is of particular interest in light of recent publications that have reported IL-12p40 polymorphism affecting age of onset and deterioration of glycemic control in certain racial groups [53,54]. Apart from this genetic link between diabetes and IL-12/IL-23p40, immunological studies have demonstrated that therapies enhancing BTLA-negative co-signalling may be a potential therapeutic target in treating autoimmune diabetes [55]. Moreover, genetic variations that encode subunits of cytokines (IL-12B, IL-23A) or cytokine receptors (IL-23R) have been associated with immune disorders such as psoriasis and psoriatic co-morbidities, including Crohn's disease and diabetes[56]. In summary, there is great scope in exploring the impact of IL-12/IL-23p40 and wound healing and its relation to several chronic diseases.

## Competing interests

The authors declare that they have no competing interests.

## Authors' contributions

MATM performed all experiments and drafted the manuscript. JMS participated in RNA extraction and flow cytometry methods. CSF provided animal samples. CSF, LWJ and SI participated in study design and manuscript writing. All authors read and approved the final manuscript.

## Supplementary Material

Additional file 1 **Table S1 - Summary of fold changes in mRNA of genes of interest**. This file contains the summary of fold changes in mRNA of all genes of interest studied. The fold changes upregulated ≥3 fold are enumerated in green, fold changes downregulated ≥3 are enumerated in red, while statistical significance ≤0.05 is enumerated in blue. Click here for file
