# Supplementary material for: Accelerated wound healing phenotype in Interleukin 12/23 deficient mice
Source: J Inflamm (Lond). 2011 Dec 20;8:39. doi: 10.1186/1476-9255-8-39 (PMC3296624; doi:10.1186/1476-9255-8-39)
Supplement: Additional file 1 — Table S1 - Summary of fold changes in mRNA of genes of interest. This file contains the summary of fold changes in mRNA of all genes of interest studied. The fold changes upregulated ≥3 fold are enumerated in green, fold changes downregulated ≥3 are enumerated in red, while statistical significance ≤0.05 is enumerated in blue. [file 1476-9255-8-39-S1.DOCX]

Additional file 1: Summary of fold changes in mRNA of genes of interest. Fold changes upregulated ≥3 fold are enumerated in green, fold changes downregulated ≥3 are enumerated in red, while statistical significance ≤0.05 is enumerated in blue.

| ymbol | **Fold Change** | ***Student's t-test*** | **Fold Change** | ***Student's t-test*** | **Fold Change** | ***Student's t-test*** | **Fold Change** | ***Student's t-test*** | **Fold Change** | ***Student's t-test*** | **Fold Change** | ***Student's t-test*** | **Fold Change** | ***Student's t-test*** | **Fold Change** | ***Student's t-test*** | **Fold Change** | ***Student's t-test*** |
| --- | --- | --- | --- | --- | --- | --- | --- | --- | --- | --- | --- | --- | --- | --- | --- | --- | --- | --- |
|  | **C57 Day 0 / C57 2hrs** | **p value** | **C57 Day 0 / C57 Day 1** | **p value** | **C57 Day 0 / C57 Day 4** | **p value** | **C57 Day 0 / C57 Day 7** | **p value** | **C57 Day 0 / p40 Day 0** | **p value** | **C57 Day 0 / p40 2hrs** | **p value** | **C57 Day 0 / p40 Day 1** | **p value** | **C57 Day 0 / p40 Day 4** | **p value** | **C57 Day 0 / p40 Day 7** | **p value** |
| Cacybp | 1.47 | 0.26 | -1.20 | **0.04** | -1.34 | **0.01** | 1.14 | 0.40 | 1.84 | **0.00** | 1.33 | 0.22 | 1.10 | 0.68 | 1.18 | 0.66 | -1.15 | 0.38 |
| Ccl1 | 1.64 | 0.21 | -1.39 | 0.21 | **-2.31** | **0.01** | -1.41 | 0.18 | **3.22** | **0.02** | 1.44 | 0.24 | 1.71 | **0.04** | 1.56 | 0.27 | **2.26** | 0.07 |
| Ccl2 | **26.74** | **0.00** | **27.21** | **0.00** | **6.02** | **0.00** | **5.16** | **0.00** | **3.05** | **0.01** | **12.07** | **0.00** | **71.59** | **0.00** | **9.62** | **0.01** | **7.92** | **0.00** |
| Ccl20 | **11.56** | **0.00** | **5.38** | **0.00** | **2.12** | 0.13 | 1.80 | 0.14 | **5.76** | **0.02** | **15.57** | **0.00** | **14.86** | **0.00** | 1.54 | 0.28 | **2.45** | **0.05** |
| Ccl22 | **3.42** | 0.16 | **4.31** | 0.06 | **2.57** | 0.14 | 1.43 | 0.70 | N/A | N/A | -1.23 | 0.87 | **9.31** | **0.02** | 1.41 | 0.70 | N/A | N/A |
| Ccl7 | **38.84** | **0.00** | **58.07** | **0.00** | **8.76** | **0.00** | **8.55** | **0.00** | **2.83** | **0.02** | **19.84** | **0.00** | **128.6** | **0.00** | **17.63** | **0.00** | **11.77** | **0.00** |
| Cd2 | 1.91 | 0.09 | -1.04 | 0.86 | -1.50 | 0.16 | 1.80 | 0.09 | 1.79 | 0.14 | **2.45** | **0.02** | 1.55 | 0.09 | 1.27 | 0.38 | 1.46 | 0.30 |
| Cd247 | -1.23 | 0.34 | -1.47 | 0.09 | -2.00 | **0.01** | **-2.04** | **0.03** | -1.42 | 0.27 | -1.63 | 0.08 | **-2.23** | **0.02** | -1.92 | **0.02** | **-2.08** | 0.06 |
| Cd28 | **-2.72** | **0.01** | **-3.18** | **0.01** | **-2.09** | **0.01** | -1.80 | **0.03** | **-3.20** | **0.00** | -1.78 | **0.01** | **-3.65** | **0.03** | -1.58 | 0.27 | **-3.20** | **0.01** |
| Cd34 | -1.76 | 0.08 | **-2.21** | **0.00** | -1.92 | **0.00** | -1.70 | **0.01** | **-2.01** | **0.01** | -1.94 | **0.00** | **-2.85** | **0.00** | -1.52 | 0.11 | -1.24 | 0.13 |
| Cd3d | 1.70 | 0.39 | 1.88 | 0.28 | 1.38 | 0.34 | **2.13** | 0.15 | -1.30 | 0.62 | 1.49 | 0.26 | **3.15** | 0.06 | **3.08** | **0.04** | -1.05 | 0.93 |
| Cd3e | 1.77 | 0.18 | **2.16** | 0.07 | 1.04 | 0.91 | **-2.04** | 0.06 | 1.33 | 0.44 | -1.14 | 0.72 | **2.06** | 0.12 | 1.09 | 0.76 | 1.67 | 0.22 |
| Cd3g | **2.81** | 0.06 | 1.50 | 0.40 | 1.39 | 0.31 | 1.58 | 0.05 | **3.38** | **0.01** | 1.76 | 0.26 | **3.96** | **0.01** | **3.89** | **0.01** | 1.02 | N/A |
| Cd4 | **2.05** | 0.09 | -1.11 | 0.77 | 1.89 | 0.08 | 1.28 | 0.62 | **2.79** | 0.05 | 1.49 | 0.31 | 1.30 | 0.36 | **2.32** | **0.03** | **3.08** | **0.05** |
| Cd40lg | 1.44 | 0.40 | -1.54 | 0.20 | **-2.83** | **0.01** | -1.30 | 0.41 | **2.90** | 0.06 | 1.30 | 0.47 | 1.47 | 0.22 | 1.40 | 0.45 | **2.03** | 0.18 |
| Cd8a | **3.00** | **0.01** | -1.03 | 0.93 | 1.08 | 0.85 | -1.37 | 0.25 | **2.84** | N/A | 1.40 | 0.29 | **3.03** | **0.02** | **2.32** | **0.04** | **4.87** | **0.03** |
| Cebpb | 1.86 | 0.09 | 1.46 | 0.05 | -1.01 | 0.95 | 1.32 | 0.16 | 1.13 | 0.46 | 1.51 | 0.07 | **2.03** | 0.06 | -1.15 | 0.77 | 1.09 | 0.60 |
| Clec7a | **3.16** | **0.01** | **10.48** | **0.00** | **9.27** | **0.00** | **2.72** | **0.00** | -1.12 | 0.34 | **2.41** | **0.00** | **26.86** | **0.00** | **5.72** | **0.00** | **7.35** | **0.00** |
| Csf2 | **8.23** | **0.00** | **2.41** | **0.01** | **2.36** | **0.02** | 1.81 | 0.10 | **2.35** | 0.05 | **2.96** | 0.08 | **43.71** | **0.00** | 1.92 | 0.19 | 1.66 | 0.20 |
| Csf3 | 1.37 | 0.47 | 1.01 | 0.97 | 1.83 | 0.17 | -1.50 | 0.25 | **3.33** | N/A | 1.19 | 0.63 | **19.19** | **0.00** | 1.46 | 0.37 | **2.12** | 0.12 |
| Cx3cl1 | 1.49 | 0.07 | 1.11 | 0.39 | -1.04 | 0.71 | 1.44 | **0.01** | 1.07 | 0.80 | 1.41 | **0.02** | 1.44 | 0.09 | 1.39 | 0.27 | 1.40 | 0.08 |
| Cxcl1 | **76.51** | **0.00** | **28.02** | **0.00** | **30.83** | **0.00** | **17.02** | **0.00** | **3.62** | **0.02** | **50.49** | **0.00** | **302.7** | **0.00** | **42.33** | **0.00** | **17.49** | **0.00** |
| Cxcl12 | -1.51 | **0.03** | 1.20 | 0.06 | 1.81 | **0.00** | 1.21 | 0.35 | -1.29 | 0.06 | -1.47 | **0.01** | 1.58 | **0.00** | 1.71 | **0.01** | 1.46 | **0.01** |
| Cxcl2 | **787.4** | **0.00** | **2073** | **0.00** | **763.9** | **0.00** | **274.5** | **0.00** | **18.27** | **0.00** | **497.9** | **0.00** | **11235** | **0.00** | **788.3** | **0.00** | **1104** | **0.00** |
| Cxcl5 | **3.72** | **0.00** | **974.4** | **0.00** | **2037** | **0.00** | **821.8** | **0.00** | **2.19** | 0.08 | 1.35 | 0.54 | **3028** | **0.00** | **1296** | **0.00** | **1523** | **0.00** |
| Il25 | 1.17 | 0.76 | -1.19 | 0.62 | **-2.56** | 0.08 | -1.31 | 0.41 | **2.79** | N/A | 1.20 | 0.61 | 1.43 | 0.22 | 1.63 | 0.25 | **2.15** | 0.14 |
| Edg1 | -1.08 | 0.82 | -1.05 | 0.62 | 1.01 | 0.89 | -1.47 | **0.02** | **-2.14** | **0.00** | -1.27 | 0.12 | -1.30 | 0.18 | -1.35 | 0.05 | -1.03 | 0.76 |
| Foxp3 | 1.53 | 0.40 | -1.19 | 0.50 | **-2.81** | **0.00** | -1.41 | 0.22 | **3.90** | **0.02** | 1.38 | 0.24 | 1.36 | 0.19 | 1.53 | 0.22 | **2.05** | 0.08 |
| Gata3 | 1.07 | 0.87 | -1.71 | 0.41 | **-2.61** | 0.15 | -1.10 | 0.85 | 1.15 | 0.83 | 1.17 | 0.76 | -1.06 | 0.91 | -1.13 | 0.84 | -1.24 | 0.75 |
| Icam1 | 1.09 | 0.90 | 1.53 | **0.02** | 1.47 | 0.09 | **-2.70** | **0.03** | **-11.93** | **0.00** | **-2.91** | **0.00** | 1.26 | 0.55 | **-2.95** | 0.18 | -1.62 | 0.14 |
| Icos | 1.07 | 0.92 | 1.19 | 0.77 | -1.15 | 0.82 | -1.02 | 0.98 | 1.18 | 0.84 | -1.34 | 0.67 | 1.20 | 0.76 | 1.74 | 0.30 | 1.58 | 0.60 |
| Ifng | 1.57 | 0.39 | -1.15 | 0.58 | **-2.59** | **0.03** | -1.32 | 0.31 | **3.38** | N/A | 1.54 | 0.29 | **2.80** | **0.04** | 1.48 | 0.40 | **2.64** | N/A |
| Il10 | **8.81** | **0.01** | **4.68** | **0.03** | **4.64** | **0.02** | **2.20** | 0.18 | 1.48 | 0.62 | **7.98** | **0.01** | **6.82** | **0.02** | **3.76** | 0.08 | **3.41** | 0.17 |
| Il12b | 1.54 | 0.27 | -1.39 | 0.21 | -1.57 | 0.36 | -1.37 | 0.19 | **3.95** | **0.01** | **3.02** | 0.06 | **6.52** | **0.01** | **5.73** | **0.00** | **9.22** | **0.00** |
| Il12rb1 | 1.46 | 0.36 | -1.39 | 0.21 | **-2.54** | **0.00** | -1.44 | 0.17 | **3.22** | **0.02** | 1.62 | 0.17 | 1.47 | 0.31 | 1.56 | 0.27 | 1.84 | N/A |
| Il12rb2 | 1.47 | 0.35 | -1.39 | 0.20 | **-2.18** | **0.04** | -1.44 | 0.16 | **3.22** | **0.02** | 1.44 | 0.24 | 1.50 | 0.12 | 1.37 | 0.47 | **2.77** | N/A |
| Il13 | 1.38 | 0.45 | **-2.06** | **0.03** | **-2.89** | 0.07 | -1.60 | N/A | **2.12** | N/A | N/A | N/A | N/A | N/A | 1.64 | N/A | 1.88 | N/A |
| Il15 | -1.38 | **0.01** | -1.39 | **0.00** | -1.15 | 0.23 | -1.36 | 0.08 | -1.07 | 0.74 | -1.30 | **0.02** | 1.03 | 0.69 | -1.00 | 0.99 | -1.14 | 0.08 |
| Il17a | **2.02** | 0.17 | 1.07 | 0.87 | **-2.26** | **0.01** | -1.28 | 0.35 | **3.63** | **0.02** | 1.82 | 0.13 | 1.69 | 0.07 | 1.75 | 0.23 | **2.54** | 0.07 |
| Il17c | 1.64 | 0.30 | -1.23 | 0.42 | **-2.26** | **0.01** | -1.34 | N/A | **3.29** | N/A | 1.49 | 0.30 | 1.69 | 0.07 | 1.75 | 0.23 | **2.07** | N/A |
| Il17d | -1.10 | 0.85 | **-2.35** | **0.03** | -1.50 | 0.14 | -1.68 | 0.12 | **-2.37** | 0.10 | -1.56 | 0.11 | -1.95 | 0.31 | -1.17 | 0.62 | -1.16 | 0.70 |
| Il17f | 1.61 | 0.43 | -1.14 | 0.72 | -1.73 | N/A | -1.23 | 0.56 | **3.94** | N/A | 1.61 | 0.27 | 1.80 | N/A | -1.11 | N/A | **3.08** | N/A |
| Il17rb | **-3.13** | **0.01** | -1.19 | N/A | **-2.59** | **0.01** | -1.80 | N/A | N/A | N/A | **-2.14** | 0.06 | **-4.27** | **0.04** | -1.20 | 0.64 | N/A | N/A |
| Il17rc | **-2.30** | 0.20 | -1.48 | 0.08 | -1.65 | 0.06 | **-4.64** | **0.00** | **-5.08** | N/A | **-5.42** | **0.00** | **-3.90** | **0.04** | **-2.99** | **0.04** | **-6.37** | **0.00** |
| Il17rd | -1.66 | 0.15 | -1.69 | **0.01** | -1.23 | 0.36 | **-2.03** | **0.01** | **-2.41** | **0.01** | **-2.23** | **0.01** | **-2.42** | **0.01** | -1.68 | **0.03** | **-2.09** | **0.04** |
| Il17re | -1.25 | N/A | **-2.40** | N/A | **-3.21** | N/A | N/A | N/A | **2.27** | N/A | -1.39 | N/A | 1.22 | N/A | -1.14 | N/A | 1.95 | N/A |
| Il18 | 1.06 | 0.75 | -1.62 | **0.00** | -1.77 | **0.00** | -1.11 | 0.40 | 1.54 | **0.01** | 1.03 | 0.83 | -1.38 | **0.05** | 1.25 | 0.46 | -1.14 | 0.20 |
| Il1b | **54.26** | **0.00** | **165.0** | **0.00** | **115.7** | **0.00** | **59.11** | **0.00** | **2.70** | **0.02** | **26.21** | **0.00** | **953.9** | **0.00** | **86.42** | **0.00** | **215.6** | **0.00** |
| Il2 | N/A | N/A | N/A | N/A | N/A | N/A | N/A | N/A | N/A | N/A | N/A | N/A | N/A | N/A | N/A | N/A | N/A | N/A |
| Il21 | 1.19 | 0.67 | -1.39 | 0.21 | **-2.54** | **0.00** | -1.44 | 0.17 | **2.93** | N/A | 1.44 | 0.24 | 1.50 | 0.12 | 1.56 | 0.27 | **2.26** | 0.07 |
| Il22 | 1.46 | 0.36 | -1.39 | 0.21 | **-2.54** | **0.00** | -1.44 | 0.17 | **3.22** | **0.02** | 1.26 | 0.45 | 1.50 | 0.12 | 1.56 | 0.27 | **2.26** | 0.07 |
| Il23a | 1.50 | 0.31 | 1.10 | 0.72 | -1.31 | 0.44 | 1.44 | 0.43 | **3.22** | **0.02** | 1.44 | 0.24 | **2.15** | 0.06 | 1.87 | 0.24 | **2.42** | 0.06 |
| Il23r | **-2.43** | **0.01** | -1.42 | 0.21 | -1.86 | 0.06 | -1.88 | **0.05** | -1.50 | 0.10 | **-2.13** | 0.22 | -1.47 | 0.16 | -1.13 | 0.49 | **-2.37** | 0.08 |
| Il27 | 1.11 | 0.78 | 1.16 | 0.75 | **-2.27** | 0.06 | -1.86 | 0.09 | 2.00 | **0.03** | -1.12 | 0.67 | 1.42 | 0.29 | 1.49 | 0.11 | 1.40 | 0.24 |
| Il3 | N/A | N/A | N/A | N/A | N/A | N/A | N/A | N/A | N/A | N/A | N/A | N/A | N/A | N/A | N/A | N/A | N/A | N/A |
| Il4 | 1.23 | 0.63 | -1.55 | 0.11 | **-3.19** | **0.00** | -1.25 | 0.19 | **2.73** | **0.02** | 1.26 | 0.38 | 1.60 | 0.24 | 1.32 | 0.51 | 1.91 | 0.08 |
| Il5 | 1.43 | 0.20 | **-2.41** | **0.03** | **-2.15** | **0.01** | -1.07 | 0.61 | 1.54 | 0.27 | 1.48 | 0.09 | **-2.26** | 0.07 | -1.02 | 0.96 | -1.15 | 0.48 |
| Il6 | **40.12** | **0.00** | **20.84** | **0.00** | **35.77** | **0.00** | **13.76** | **0.00** | 1.40 | N/A | **5.18** | **0.00** | **247.1** | **0.00** | **24.59** | **0.00** | **14.54** | N/A |
| Il6ra | -1.95 | 0.10 | -1.38 | 0.07 | -1.85 | **0.01** | **-2.48** | **0.00** | **-2.25** | **0.02** | **-2.30** | **0.00** | -1.77 | **0.01** | **-2.18** | **0.03** | -1.92 | **0.04** |
| Il7r | -1.71 | 0.32 | **6.98** | **0.00** | **3.48** | **0.00** | 1.21 | 0.56 | -1.09 | N/A | -1.88 | 0.08 | **6.95** | **0.00** | 1.69 | 0.18 | **2.21** | 0.10 |
| Isg20 | 1.46 | 0.36 | -1.39 | 0.21 | **-2.54** | **0.00** | -1.44 | 0.17 | **3.22** | **0.02** | 1.44 | 0.24 | 1.50 | 0.12 | 1.56 | 0.27 | **2.26** | 0.07 |
| Jak1 | 1.11 | 0.53 | -1.07 | 0.45 | -1.02 | 0.88 | 1.39 | **0.05** | 1.26 | 0.21 | 1.53 | **0.03** | 1.24 | 0.25 | 1.30 | 0.28 | -1.06 | 0.67 |
| Jak2 | -1.21 | 0.32 | -1.85 | 0.18 | 1.00 | 0.98 | 1.22 | 0.29 | -1.03 | 0.93 | -1.18 | 0.44 | 1.14 | 0.64 | 1.20 | 0.46 | -1.26 | 0.41 |
| Mmp13 | 1.31 | 0.53 | **51.07** | **0.00** | **530.3** | **0.00** | **65.24** | **0.00** | **2.90** | **0.01** | 1.90 | 0.10 | **57.64** | **0.00** | **119.8** | **0.00** | **262.7** | **0.00** |
| Mmp3 | 1.39 | 0.19 | **19.75** | **0.00** | **91.90** | **0.00** | **10.58** | **0.00** | -1.42 | 0.26 | 1.79 | **0.01** | **33.31** | **0.00** | **11.81** | **0.00** | **20.83** | **0.00** |
| Mmp9 | **5.34** | **0.00** | **13.46** | **0.00** | **22.87** | **0.00** | **8.02** | **0.00** | 1.21 | 0.51 | **5.32** | **0.00** | **33.55** | **0.00** | **7.72** | **0.00** | **21.07** | **0.00** |
| Myd88 | -1.70 | 0.35 | 1.44 | **0.02** | 1.24 | 0.28 | **-2.87** | **0.01** | **-3.22** | **0.00** | **-5.33** | **0.00** | -1.47 | 0.37 | **-2.31** | 0.07 | **-2.81** | **0.01** |
| Nfatc2 | **-2.14** | 0.12 | **-2.90** | **0.00** | -2.00 | **0.00** | -1.87 | **0.00** | -1.86 | **0.03** | -1.61 | 0.05 | **-2.80** | **0.01** | -1.85 | 0.07 | -1.79 | **0.00** |
| Nfkb1 | -1.15 | 0.28 | -1.17 | **0.01** | -1.06 | 0.09 | -1.05 | 0.28 | -1.27 | 0.10 | -1.33 | **0.00** | 1.08 | 0.59 | -1.10 | 0.36 | -1.06 | 0.35 |
| Rorc | -1.96 | 0.06 | -1.34 | **0.01** | **-4.74** | **0.00** | **-3.71** | **0.00** | **-2.78** | **0.00** | **-2.37** | **0.00** | -1.97 | 0.08 | **-3.46** | **0.01** | **-2.29** | **0.01** |
| Socs1 | -1.58 | 0.29 | -1.28 | 0.39 | -1.58 | 0.08 | **-2.90** | **0.02** | -1.29 | 0.50 | **-2.65** | **0.02** | 1.04 | 0.85 | **-2.10** | **0.02** | 1.17 | 0.65 |
| Socs3 | 1.55 | 0.57 | 1.69 | **0.03** | 1.60 | 0.07 | **-3.86** | **0.01** | **-5.82** | N/A | **-4.56** | **0.01** | **3.34** | **0.02** | **-2.39** | 0.11 | 1.06 | 0.85 |
| Stat3 | **-4.03** | 0.09 | -1.04 | 0.70 | -1.63 | **0.01** | **-4.73** | **0.00** | **-12.38** | **0.00** | **-9.26** | **0.00** | -1.98 | 0.20 | **-4.99** | 0.06 | **-3.53** | **0.00** |
| Stat4 | 1.46 | 0.36 | -1.07 | 0.87 | **-2.54** | **0.00** | -1.44 | 0.17 | **3.22** | **0.02** | 1.40 | 0.38 | 1.50 | 0.12 | 1.56 | 0.27 | **2.26** | 0.07 |
| Stat5a | -1.60 | 0.23 | 1.34 | 0.08 | 1.06 | 0.68 | -1.05 | 0.77 | -1.91 | **0.03** | -1.45 | 0.14 | -1.04 | 0.88 | -1.26 | 0.49 | -1.66 | **0.05** |
| Stat6 | -1.50 | 0.19 | -1.04 | 0.86 | -1.11 | 0.69 | -1.19 | 0.47 | **-2.58** | 0.05 | **-2.35** | 0.05 | -1.96 | 0.06 | -1.54 | 0.19 | -1.90 | 0.13 |
| Syk | -1.57 | 0.16 | 1.26 | 0.52 | 1.07 | 0.77 | **-2.18** | 0.06 | -1.90 | 0.12 | **-3.29** | **0.01** | -1.03 | 0.94 | -1.29 | 0.53 | **-2.17** | 0.07 |
| Tbx21 | 1.43 | 0.38 | -1.42 | 0.16 | **-2.60** | **0.00** | -1.46 | 0.13 | **3.16** | **0.02** | 1.41 | 0.24 | 1.47 | 0.11 | 1.35 | 0.49 | **2.22** | 0.06 |
| Tgfb1 | **-3.79** | 0.09 | 1.48 | 0.06 | 1.14 | 0.53 | -1.82 | **0.04** | **-5.79** | **0.00** | **-2.38** | **0.00** | 1.17 | 0.50 | -1.46 | 0.23 | -1.16 | 0.66 |
| Tirap | **-2.17** | **0.02** | -1.08 | 0.62 | -1.30 | 0.10 | -1.34 | 0.07 | -1.63 | 0.07 | **-2.13** | **0.01** | -1.40 | 0.09 | -1.18 | 0.30 | -1.62 | 0.08 |
| Tlr4 | -1.41 | 0.61 | -1.19 | 0.66 | -1.73 | 0.23 | -1.02 | 0.96 | -1.07 | 0.94 | **-2.01** | 0.24 | 1.14 | 0.81 | -1.26 | 0.62 | -1.47 | 0.56 |
| Tnf | 1.54 | 0.27 | **10.98** | **0.00** | 1.61 | 0.09 | 1.00 | 0.99 | **2.93** | N/A | 1.44 | 0.24 | **4.89** | **0.00** | 1.07 | N/A | **2.26** | 0.07 |
| Traf6 | **-2.36** | 0.18 | -1.33 | 0.14 | -1.47 | 0.07 | **-2.92** | **0.00** | **-7.57** | **0.01** | **-4.28** | **0.00** | **-2.66** | 0.10 | **-2.78** | 0.10 | **-3.09** | **0.02** |
| Yy1 | 1.64 | 0.23 | -1.13 | 0.22 | -1.46 | **0.00** | 1.27 | 0.19 | **2.37** | **0.00** | 1.65 | 0.07 | 1.41 | 0.33 | 1.49 | 0.39 | 1.73 | **0.04** |
| Gusb | 1.04 | 0.79 | 1.64 | **0.00** | **2.13** | **0.00** | 1.90 | **0.00** | 1.06 | 0.77 | 1.10 | 0.40 | 1.44 | **0.02** | 1.83 | **0.00** | 1.66 | **0.05** |
| Hprt1 | 1.88 | 0.16 | -1.20 | **0.01** | -1.36 | **0.00** | 1.36 | 0.08 | **3.06** | **0.00** | 1.81 | **0.02** | 1.31 | 0.31 | 1.68 | 0.20 | 1.20 | 0.06 |
| Hsp90ab | 1.56 | 0.20 | -1.07 | 0.32 | -1.24 | **0.01** | 1.45 | **0.04** | 1.98 | **0.00** | 1.95 | **0.00** | 1.34 | 0.25 | 1.20 | 0.57 | 1.23 | 0.12 |
| Gapdh | **-2.06** | 0.13 | -1.67 | **0.02** | -1.82 | **0.01** | **-2.40** | **0.02** | **-2.36** | **0.02** | **-2.05** | **0.02** | **-2.32** | **0.05** | **-2.21** | 0.07 | -1.89 | 0.05 |
| Actb | -1.49 | 0.37 | 1.30 | **0.00** | 1.43 | **0.00** | -1.56 | **0.02** | **-2.73** | **0.00** | -1.88 | **0.01** | -1.09 | 0.71 | -1.67 | 0.24 | -1.30 | **0.01** |
